# Neuromuscular diseases associated with COVID-19 vaccines: a systematic review and pooled analysis of 258 patients

**DOI:** 10.1186/s12883-023-03486-y

**Published:** 2023-12-11

**Authors:** Amirhossein Tayebi, Parham Samimisedeh, Elmira Jafari Afshar, Saeideh Mahmoudnia, Nesa Milan, Aryan Ayati, Aryan Madady, Hadith Rastad

**Affiliations:** 1 Cardiovascular Research Center, Alborz University of Medical Sciences, Karaj, Iran; 2 Department of Neurology, Shahid Rajaei Hospital, Alborz University of Medical Sciences, Karaj, Iran; 3 Center of Orthopedic Trans-Disciplinary Applied Research (COTAR), Department of Orthopedics, Tehran university of medical sciences, Tehran, Iran; 4 Tehran University of Medical Sciences (TUMS), Tehran, Iran

**Keywords:** COVID-19 vaccine, Peripheral neuropathy, Neuromuscular diseases, Guillain-Barré syndrome, Parsonage-turner syndrome, Myasthenia gravis, Facial nerve palsy, Small fiber neuropathy, Tolosa-Hunt syndrome

## Abstract

**Background:**

Neuromuscular diseases (NMD) emerged as one of the main side effects of the COVID-19 vaccination. We pooled and summarized the evidence on the clinical features and outcomes of NMD associated with COVID-19 vaccination.

**Methods:**

We comprehensively searched three databases, Medline, Embase, and Scopus, using the key terms covering “Neuromuscular disease” AND “COVID-19 vaccine”, and pooled the individual patient data extracted from the included studies.

**Results:**

A total of 258 NMD cases following COVID-19 have been reported globally, of which 171 cases were Guillain-Barré syndrome (GBS), 40 Parsonage-Turner syndrome (PTS), 22 Myasthenia Gravis (MG), 19 facial nerve palsy (FNP), 5 single fiber neuropathy, and 1 Tolosa-Hunt syndrome. All (100%) SFN patients and 58% of FNP patients were female; in the remaining NMDs, patients were predominantly male, including MG (82%), GBS (63%), and PTS (62.5%).

The median time from vaccine to symptom was less than 2 weeks in all groups. Symptoms mainly appeared following the first dose of vector vaccine, but there was no specific pattern for mRNA-based.

**Conclusion:**

COVID-19 vaccines might induce some NMDs, mainly in adults. The age distribution and gender characteristics of affected patients may differ based on the NMD type. About two-thirds of the cases probably occur less than 2 weeks after vaccination.

**Supplementary Information:**

The online version contains supplementary material available at 10.1186/s12883-023-03486-y.

## Highlights

• Neuromuscular diseases (NMD) have emerged as a significant side effect of COVID-19 vaccination.

• The study provides a comprehensive summary of the clinical features and outcomes of NMD associated with COVID-19 vaccination.

• A total of 258 cases of NMD following COVID-19 vaccination were reported globally.

• The most commonly reported NMDs were Guillain-Barré syndrome (GBS) and Parsonage-Turner syndrome (PTS).

• Symptoms typically appeared within 2 weeks after vaccination, with a higher likelihood following the first dose of vector vaccine.

**Supplementary Information:**

The online version contains supplementary material available at 10.1186/s12883-023-03486-y.

## Introduction

In the long term, vaccination remains vital in the COVID-19 pandemic control strategy [1]. More than 13.3 billion COVID-19 vaccines have been administered globally; however, the fading of immunity over time necessitates booster doses; hence, about 1 million shots are still administered daily worldwide [2].

However, the extraordinary speed of vaccine production and the waiver of essential testing steps raised hesitancy regarding the vaccines’ safety [3–5]. A wide range of side effects are reported worldwide shortly after vaccination. While most side effects of vaccines are mild, their impact on the central and peripheral nervous systems often necessitates medical interventions [6–10].

Recently, increasing case series/reports have characterized the clinical course in patients with some types of peripheral neuropathy (Guillain-Barré syndrome, Parsonage-Turner syndrome, Small fiber neuropathy, and Tolosa-Hunt syndrome), cranial neuropathy (Facial nerve palsy), and neuromuscular junction disorder (Myasthenia gravis) [11] following COVID-19 vaccination. In this study, we pooled and summarized available evidence to enhance the knowledge of the course and prognosis of the neuromuscular diseases associated with COVID-19 vaccines, which can lead to a well-timed diagnosis and management of these patients.

## Method

We conducted this systematic review in accordance with the Preferred Reporting Items for Systematic Reviews and Meta-Analyses (PRISMA) statement. Patient consent or ethical approval was not required due to the review nature of the study. All studies reporting the symptoms and clinical course of patients with neuromuscular disease (NMD) associated with the COVID-19 vaccine were included.

### Search

We comprehensively searched three databases, Scopus, Medline, and Embase, on 25 January 2023 and updated on 20 February 2023, using a combination of the key terms in multiple domains, including “COVID-19 vaccine”, “Peripheral neuropathies”, “Cranial neuropathy”, “neuromuscular disease”. Also, we checked the reference lists and citing publications of the included articles for any additional eligible studies. We imported all retrieved citations into Zotero 6.0.20 and removed the duplicate items.

### Inclusion process and criteria

Two researchers [AT and PS] independently screened the titles, abstracts, and full texts of the studies to include the eligible articles. All studies meeting our eligibility criteria were included:

1. Reporting new-onset case (s) of any NMD following COVID-19 vaccination
2. An observational design: case series, case reports, and cohort studies
3. Written in English

### Data extraction

Three researchers [EJ, SM, NM] extracted the data from the retrieved articles into a predefined form in Microsoft Excel (version 2019, Microsoft Corp., Redmond, WA, USA). The extracted data included the first author’s name, year, country, age, gender, clinical data, type of the NMD, subtype of the disease, vaccine type, vaccine dose number (first, second, booster), the time between injection and symptom onset, presenting signs and symptoms, electrophysiological findings, laboratory data, treatment, and patients follow up.

### Quality assessment

The quality of the included case series was assessed independently by two trained researchers (AA and SM) using the Joanna Briggs Institute (JBI) appraisal tool adapted for case series [12]; a third researcher (AT or HR) resolved any disagreement. JBI appraisal tool included 10 items, each receiving one score (Supplementary Table 1).

### Statistical analyses

To organize the extracted individual patient data (IPD) and to produce tables, we used Microsoft Excel (version 2019, Microsoft Corp., Redmond, WA, USA). Based on the disease, we categorized integrated IPD case series and case reports into a relevant case series. After excluding cases without data for the characteristic of interest, we calculated a valid percent for each categorical and a median (Interquartile range) or mean and standard deviation (SD) for continuous variables. Data analysis was conducted using Stata/MP Version 16 (Stata Corp. LP, USA/ METAN package).

## Results

After excluding duplicates (*n* = 244) and ineligible items from the 879 publications identified in the initial search, 133 case reports/case series met our eligibility criteria. All included studies had a quality score greater than 7/10 (Supplementary Table 1).

**Fig. 1:**
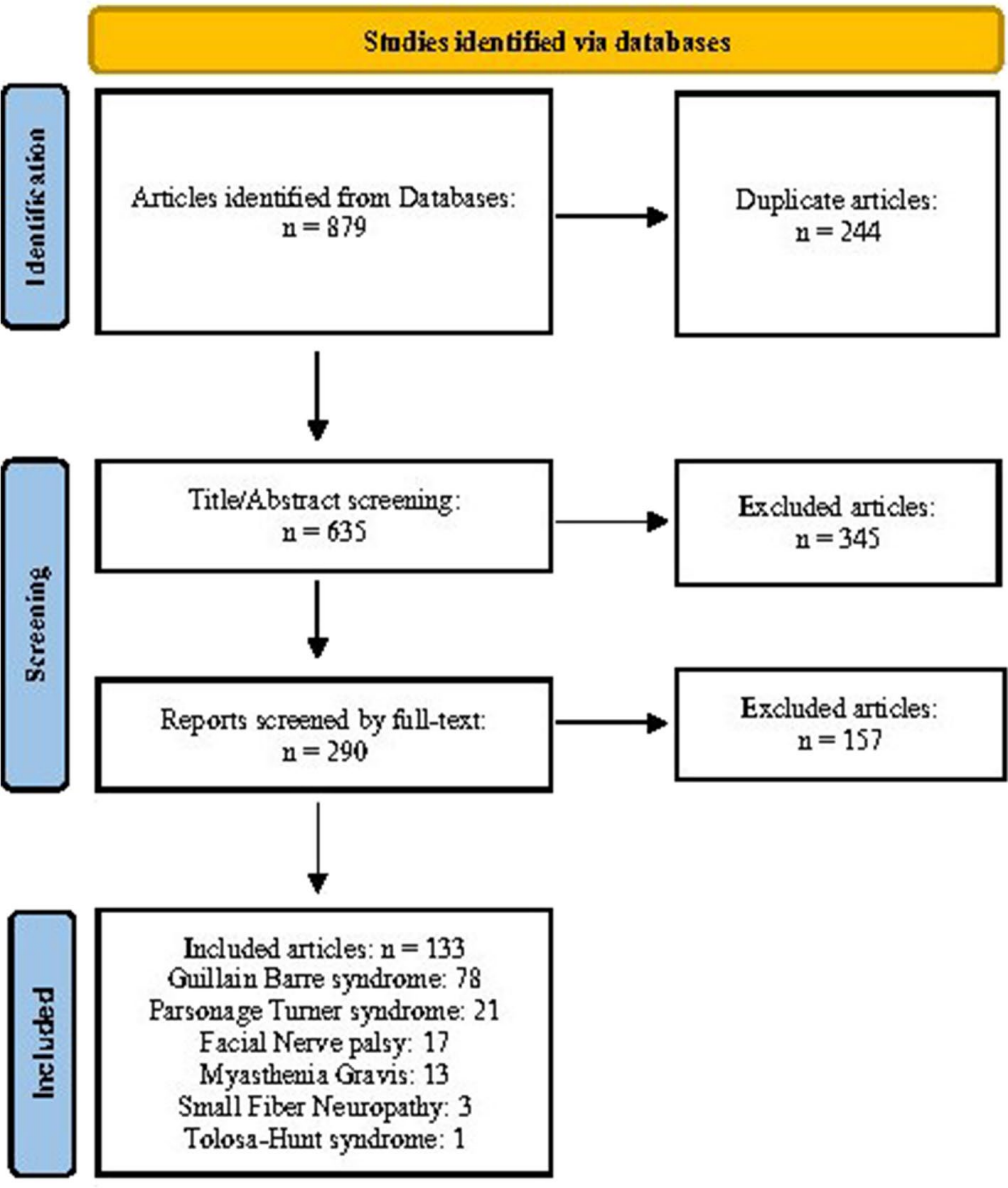
PRISMA flow chart of the eligible study selection

Based on our pooled analyses, a total of 258 cases of NMD associated with COVID-19 vaccines have been reported globally. In order by frequency, the type of NMD was Guillain-Barré syndrome (GBS, *n* = 171), Parsonage-Turner syndrome (PTS, *n* = 40), Myasthenia gravis (MG, *n* = 22), Facial nerve palsy (FNP, *n* = 19), Small fiber neuropathy (SFN, *n* = 5), and Tolosa-Hunt syndrome (*n* = 1) (Fig. 1). Reported cases were mainly adults (98%) aged 18 years or older, and in more than two-thirds of them, the symptoms appeared less than 2 weeks after the vaccination.

### Guillain-Barré syndrome

Seventy-eight articles (57 case reports [13–69] and 21 case series [70–90]) containing 171 COVID-19 vaccine-associated GBS patients were reported. About half of the cases were reported from South Korea (*n* = 21), Italy (*n* = 20), The USA (*n* = 18), and Australia (*n* = 18) (Supplementary Tables 2 and 3).

The reported cases were aged between 14 and 90 years old, with a median (IQR) of 58 [43, 70] years, and 108 (63%) of cases were male. Regarding vaccine type, 31% (52/168) of patients received an mRNA and 69% (116/168) a vector-based vaccine. In most cases vaccinated with vector-based vaccines, symptoms appeared after the first dose (First/ Second doses (n): 105/ 11); however, such a pattern was not observed in cases vaccinated with mRNA-based vaccines (First/ Second/ Booster doses (n): 34/ 16/ 2). The median (IQR) time from vaccine to symptoms was 13 [8, 17] days, ranging from 1 to 44 days. Regarding GBS pathogenic subtypes, 81% (77/95) of patients had acute inflammatory demyelinating polyneuropathy, 12% (11/95) acute motor axonal neuropathy, and 7% (7/95) acute motor and sensory axonal neuropathy. GBS clinical variants and pathogenic subtypes are summarized in Table 1.

**Table 1:** Characteristics, findings, treatments, and clinical outcome of reported polyneuropathy cases

Variable	Sample size		Median (IQR) / n (%)
***Guillain Barre syndrome***			
Age (year), Median (IQR)		171	58 (43, 70)
Gender, n (%) male			108 (63%)
mRNA-based vaccine^a^	1st dose / 2nd dose / Booster	52	34 (65%) / 16 (31%) / 2 (4%)
Vector-based vaccine^b^	1st dose / 2nd dose	116	105 (91%) / 11 (9%)
Clinical variant	BFP / Typical / MFS / SM / Paraparetic / FDFN	58	16(28%)/12(21%)/9(16%)/9(16%)/7 (12%)/5(9%)
Pathogenic subtype	AIDP / AMAN / AMSAN	95	77 (81%) / 11(12%) / 7(7%)
Time from vaccine to symptom (days), Median (IQR)		171	13 (8, 17)
**Tests**			
Diagnostic MRI		69	20 (29%)
Diagnostic EMG / NCS		135	131 (97%)
Laboratory tests	Positive Albuminocytological dissociation	123	105 (85%)
	Positive Anti ganglioside Antibodies	70	17 (24%)
**Treatment**			
IVIG only / + PLEX / or + Corticosteroids		142	107 (75%) / 10 (7%) / 7 (5%)
PLEX only / Corticosteroid only / Conservative			9 (6%) / 4 (3%) / 4 (3%)
**Clinical outcomes**			
Complete / Partial / or Poor recovery at follow-up / or Death		123	65 (53%) / 46 (37%) / 11 (9%) / 3 (2%)
***Parsonage-turner syndrome***			
Age (year), Median (IQR)		40	50 (38, 63)
Gender, n (%) male			25(62.5%)
mRNA-based vaccine^a^	1st dose / 2nd dose	28	14 (50%) / 14 (50%)
Vector-based vaccine^b^	1st dose / 2nd dose	12	10 (83%) / 2 (17%)
Symptom side	Injection side / Contralateral / Bilateral	38	30 (79%) / 5 (13%) / 3 (%)
Presenting symptoms	Shoulder or arm pain / Weakness / Paresthesia	28	20 (71%) / 17 (61%) / 8 (29%)
Time from vaccine to symptom (days), Median (IQR)		40	8 (5, 15)
**Tests**			
Positive EMG / NCS		26	22 (85%)
Positive Brachial plexus MRI		35	11 (31%)
**Treatment**			
Corticosteroid without / with GABA analogue		38	14 (37%) / 10 (26%)
NSAID + GABA analogue			2 (5%)
IVIG			1 (2.5%)
**Clinical outcomes**			
Complete / Partial / or Poor recovery at follow-up		37	15 (41%) / 15 (41%) / 7 (18%)
***Facial nerve palsy***			
Age (year), Median (IQR)		19	38 (34, 57)
Gender, n (%) male			8 (42%)
Prior history of facial nerve palsy			4 (21%)
mRNA-based vaccine^a^	1st dose / 2nd dose	14	11 (79%) / 3 (21%)
Vector-based vaccine^b^	1st dose / 2nd dose	5	4 (80%) / 1 (20%)
Presenting symptoms	Ipsilateral / Bilateral facial weakness	19	17 (90%) / 2 (10%)
Time from vaccine to symptom (days), Median (IQR)		19	3 (2, 6)
**Treatment**			
Corticosteroids only/ + viral agents / + Fluorometholone / or + topical antibiotics		18	10 (56%) / 6 (33%) / 1 (6.5%) / 1 (6.5%)
**Clinical outcomes**			
Complete recovery		17	17 (100%)
***Small fiber neuropathy***			
Age (year), Median (IQR)		5	52 (36, 60.5)
Gender, n (%) male			0
mRNA-based vaccine^a^	1st dose / 2nd dose / Booster		0 / 4 (80%)/ 1 (20%)
Time from vaccine to symptom (days), Median (IQR)			10 (4, 19)
Presenting symptoms	Weakness/gait disturbances/dysesthesia/paresthesia/dysphagia		2(40%)/2(40%)/2(40%)/1(20%)/1(20%)
**Tests**	Diagnostic skin Punch biopsy	4	4 (100%)
**Treatment**	Symptom treatment / corticosteroid + IVIG	4	3 (75%)/ 1 (25%)
**Outcome**	Resolved in less than two weeks	3	3 (100%)

*AIDP* Acute inflammatory demyelinating polyneuropathy, *AMAN* Acute motor axonal neuropathy, *AMSAN* Acute motor and sensory axonal neuropathy, *BFP* Bilateral facial palsy, *CT* Computed Tomography, *EMG* Electromyography, *F* Female, *FDFN* Focal demyelination of facial nerves, *FW* Facial Weakness, *GABA* Gamma-Aminobutyric Acid, *IM* Intramuscular, *IQR* Interquartile range, *IVIG* Intravenous Immunoglobulin, *J&J* Johnson and Johnsons, *MRI* Magnetic Resonance Imaging, *M* Male, *MFS* Miller fisher syndrome, *mRNA* messenger Ribonucleic Acid, *NCS* Nerve conduction study, *NR* Not reported, *NSAID* Non-steroidal anti-inflammatory drug, *PLEX* Plasma Exchange, SM Sensory motor

^a^Pfizer or Spikevax, ^b^AstraZeneca, Covaxin, Sputnik V, or Johnson and Johnson

Brain MRI was diagnostic for GBS in only 20 out of 69 patients (sensitivity: 29%); however, Electromyogram / Nerve conduction study (EMG / NCS) was diagnostic in almost all cases (131/135, 97%). Regarding laboratory tests, Albuminocytological dissociation and Anti-ganglioside antibodies were positive in 85% (105/123) and 24% (17/70) of patients, respectively. Based on the reported data, most patients were treated with IVIG only (107/142, 75%); 7% (10/142) underwent IVIG plus plasma exchange treatment, and 6% (9/160) received plasma exchange alone (Table 1). At the follow-up, complete or partial recovery was noted for most patients (65/123 (53%) and 46/123 (37%), respectively). However, death occurred in 3 patients (2%), and 11 out of 123 patients (9%) had a poor recovery.

### Parsonage-turner syndrome

A total of 40 patients with PTS associated with COVID-19 vaccines [77, 91–110]. Most cases were reported from The USA (*n* = 15) and South Korea (*n* = 13) (Table 1, Supplementary Table 4).

The median (IQR) age was 50 [38, 63] years, ranging from 14 to 84 years, and 25 (62.5%) patients were male. Regarding vaccine type, 70% of cases (*n* = 28) received mRNA-based vaccines. While in most of the patients with vector vaccine-associated PTS, the symptoms appeared on the first dose of the vaccine (10/12), such a pattern was not observed in mRNA vaccine-related PTS (First/Second doses (n): 14/14).

Of the 38 cases reporting the symptoms localizations, 30 (79%) experienced symptoms ipsilaterally on the injection side, 5 contralateral to the injection side, and 3 bilateral. The presenting symptoms were shoulder or arm pain (20/28), muscle weakness (17/28), and paresthesia (8/28). The median (IQR) interval from vaccine injection to symptoms was 8 [5, 15] days, ranging from 1 to 56 days. EMG / NCS was diagnostic in 85% (22/26) of the patients, and brachial plexus MRI in 31% (11/35). The follow-up duration varied among the studies. However, a complete recovery was noted in 41% (15/37) of patients, a partial recovery in 41% (15/37), and a poor improvement in symptoms in 18% (7/37) (Table 1, Supplementary Table 5).

### Facial nerve palsy

Nineteen FNP cases were reported following COVID-19 vaccination [111–127] across the world. The patients’ ages ranged from 17 to 62 years (median (IQR): 38 [34, 57] years), 11 out of 19 patients were female (58%), and 4 patients had a prior history of FNP. In 14 patients, the symptoms occurred after receiving mRNA-based (Pfizer or Spikevax) and 5 after vector-based vaccines (AstraZeneca, Covaxin, Sputnik V, or Johnson and Johnson). Symptoms appeared mainly after the first dose in mRNA- and vector-based vaccine-associated FNP patients. Almost all patients had an ipsilateral FNP (17/19). The time interval from vaccine injection to symptoms ranged from 1 to 18 days, with a median (IQR) of 3 [2, 6] days (Table 1, Supplementary Table 6).

Regarding medications, 10 (56%) patients were treated with corticosteroids alone, 6 (33%) with both corticosteroids and a viral agent. Corticosteroids were combined with Fluorometholone eye drops in one case and with a topical antibiotic in another patient. At the follow-up, the symptoms had resolved in all patients without any complications.

### Small fiber neuropathy

We included three articles (two case reports [128, 129] and one case series [130]) reporting five COVID-19-associated small fiber neuropathy (SFN) in our study (Table 1, Supplementary Table 7). These patients were reported from Austria (*n* = 3) and the USA (*n* = 2). All five patients were female, aged between 32 and 64, and had received mRNA vaccines (Pfizer and Spikevax). In 4 out of 5 patients, symptoms appeared after receiving the second dose.

The presenting symptoms were weakness (2/5), gait disturbances (2/5), dysesthesia (2/5), paresthesia (1/5), and dysphagia (1/5). Skin punch biopsy helped the diagnosis in all cases (4/4). Treatment was reported in 4 patients. Except for patient #1, who was treated with corticosteroid and IVIG, the other patients only received symptomatic therapies. The outcome was reported in 3 of the patients; symptoms resolved in all three in less than 2weeks (Table 1).

### Tolosa-hunt syndrome

One Tolosa-Hunt patient from the USA was included in our study [131] (Supplementary Table 7). The patient was a 45-year-old male who received a vector-based vaccine. The patient presented with left-sided headache, periorbital pain, ptosis, and decreased visual acuity. The brain CT indicated a sinus thrombosis, while the brain MRI reported a perineural enhancement surrounding the optic nerve sheath. The patient underwent steroid treatment, and the symptoms improved in 2 months.

### Myasthenia gravis

Twenty-two Myasthenia Gravis (MG) patients associated with COVID-19 vaccines were reported from eight countries [8, 132–143], more frequently from the UK (*n* = 7) and Italy (*n* = 5) (Supplementary Tables 8 and 9).

Patients age ranged from 13 to 90 years (median (IQR): 64 [50, 74] years) and 18 of them were male (18/22). Most patients (14/22) received an mRNA-based vaccine (Pfizer-BioNTech or Spikevax). Vector-based vaccine receivers developed symptoms mainly after the first dose (7/8); however, such a pattern was not observed in patients who received mRNA-based vaccines (First/ second/ third doses (n): 5/ 5/ 4, Table 2).

**Table 2:** Characteristics, findings, treatments, and clinical outcome of reported Myasthenia gravis cases

Variable		Sample size	n (%)
Age (year), Median (IQR)		22	64 (50, 74)
Gender, n (%) male			18 (82%)
Generalized Myasthenia Gravis			12 (55%)
mRNA-based vaccine^a^	1st dose / 2nd dose / Booster	14	5 (36%) / 5 (36%) / 4 (28%)
Vector-based vaccine^b^	1st dose / 2nd dose	8	7 (88%) / 1 (12%)
Presenting symptoms	Diplopia / Ptosis^c^ / Dysarthria / Dysphagia / Extremity asthenia	13	10(77%)/ 6(46%)/ 3(23%)/ 3(23%)/ 1(8%)
Time from vaccine to symptom (days), Median (IQR)		22	6 (2, 8.5)
**Tests**			
Imaging	Normal Brain CT scan	5	5 (100%)
	Normal Brain MRI	6	5 (83%)
	Thymus hyperplasia on Chest CT scan	8	2 (25%)
Tests in the clinic	Positive Ice pack test	2	2 (100%)
	Positive IM neostigmine test	4	3 (75%)
EMG	Positive RNS	15	13 (87%)
	Positive SFEMG	4	4 (100%)
Seropositivity	Only AChR Ab positive (MuSK not tested)	16	14 (87%)
	AChR positive & MuSK negative	4	2 (50%)
	AChR negative & MuSK negative		2 (50%)
**Treatment**			
Pyr only / + Pred / + IVIG / or + PLEX		19	7 (33%) / 7 (33%) / 1 (5%) / 1 (5%)
Pyr + Pred + IVIG / Pyr + Pred + PLEX / Pred + PLEX			2 (10%) / 2 (10%) / 1 (5%)
**Clinical outcomes**			
Partial / Complete improvement at discharge or 1-month follow-up		14	7 (50%) / 4 (29%)
Myasthenic crisis/symptoms unchanged at 3-months follow-up			2 (14%) / 1 (7%)

*AChR* Acetylcholine Receptor antibody, *CT* Computed Tomography, *EMG* Electromyography, *IM* Intramuscular, *IQR* Interquartile range, *IVIG* Intravenous Immunoglobulin, *MRI* Magnetic Resonance Imaging, *mRNA* messenger Ribonucleic Acid, *MuSK* Muscle Specific Kinase antibody, *PLEX* Plasma Exchange, *Pred* Prednisolone, *Pyr* pyridostigmine, *RNS* Repetitive Nerve Stimulation, *SFEMG* Single Fiber Electromyography

^a^Pfizer or Spikevax, ^b^AstraZeneca, ^c^all bilateral except patient #18, who had left-sided ptosis

The presenting symptoms were binocular diplopia (10/13), ptosis (6/13), dysarthria (3/13), dysphagia (3/13), and/or weakness of the lower or upper limbs (1/13). The median (IQR) time interval from vaccine injection to symptoms was 6 (2, 8.5) days. In terms of MG type, 10 patients had an ocular or bulbar MG, and the rest generalized MG (Table 2).

Imaging evaluations were performed for some patients to rule out other causes, including brain CT scan (abnormal findings: 0/5), brain MRI (abnormal findings: 1/6), chest CT scan (abnormal findings: 2/8, Thymoma or thymic hyperplasia) (Table 2, Supplementary Table 9).

Repetitive nerve stimulation (RNS) was positive in 13 out of 15, and Single fiber electromyography (SFEMG) in all 4 patients who received the test. Acetylcholine receptor (AChR) antibody was positive in 16 out of 20 patients and Muscle-specific kinase (MuSK) negative in all 4 tested patients. Complete improvement at discharge/1-month follow-up was reported in 79% (11/14), a myasthenic crisis in 14% (2/14), and unchanged symptoms at 3 months in 7% (1/14) of the cases.

## Discussion

Based on our comprehensive literature review, a total of 171 GBS, 40 PTS, 22 MG, 19 FNP, 5 SFN, and 1 Tolosa-Hunt syndrome cases following COVID-19 vaccination have been reported so far across the world. Overall, reported cases were mainly adults; FNP patients (median age: 38 years) were the youngest, and MG patients (64 years) were the oldest group. While all SNF and most FNP cases were female, in other groups, the male gender was predominant. The median time from vaccine to symptom was less than 2 weeks in all groups, ranging from 3 days in FNP patients to 13 days in GBS cases. Symptoms mainly appeared following the first dose of vector-based vaccine, but there was no specific pattern for mRNA-based associated NMD. While most reported cases experienced a benign course of the disease, death occurred in 3 out of 123 GBS patients, and 2 out of 14 MG patients developed a myasthenic crisis.

The occurrence of NMD has been reported following influenza and other vaccines [133, 144–148]. A pooled analysis of GBS following mass immunizations with Influenza, Human papillomavirus, and Measles-Rubella vaccines, including 58 studies (2,110,441,600 participants), revealed an incidence rate of 3.09 per million in 6 weeks of vaccination, without a sex difference. Contrary to GBS induced by COVID-19 vaccines, most GBS cases associated with other vaccines were younger than 18 or older than 60 years old; their different target population could explain this dissimilarity. Furthermore, based on the CDC’s vaccine adverse events reporting system (VAERS) database, 944 individuals developed FNP after other vaccines in the United States between 2009 and 2018 [149]. A little more than half of the patients were female (55.7%), and only 2.2% had a prior FNP history. A similar gender pattern was observed in FNP following COVID-19 vaccines. However, a prior history of FNP was reported in 21% of patients.

Also, a total of 42 definite incident cases of MG occurred following Influenza and Hepatitis B vaccines in American adults between 1990 and 2017 [150]; these cases had a higher mean (SD) time from vaccine to symptoms compared to MG cases associated with COVID-19 vaccines (10 (10.7) vs 7.1(6.6), respectively). Overall, MG is mainly diagnosed between 10 and 70 years old [23, 24], and affected female patients are younger than their male counterparts (mean age: 28 vs. 42 years, respectively) [25]. Gender ratio differs by MG type; contrary to pure ocular or bulbar MG, cases with generalized MG are predominantly female (female/male ratio: 3:2 or higher) [26]. Less than 40% of MG cases following other vaccines were older than 60 years (Mean age (SD): 49.0 (18.9) years, Range: 18 to 84 years), without a gender predominance [22]. However, COVID-19 vaccine-associated MG cases were predominantly male, overall, and by MG type, and half of them were aged 70 years or older (Mean age (SD): 61.0 (19.3) years).

In this study, we summarized data on COVID-19 vaccine-associated NMD provided by case reports/series. The exact mechanism by which vaccines could induce neuromuscular diseases remains unknown. However, the immunization processes following the vaccines could provoke an autoimmune response by establishing an inflammatory environment. If vaccine antigens mimic self-antigens, the immune response could cross-react with self-antigens, leading to an autoimmune reaction (Molecular mimicry mechanism). Furthermore, during immune responses to vaccine antigens, inflammatory signals can activate self-reactive T cells involved in the autoimmune processes (Bystander effect mechanism). Also, the activation of self-reactive T cells could be triggered via self-antigens released upon self-tissue damage following inflammatory cascade (Epitope spreading mechanism). Vaccines containing dsRNA or its analogs can also overexpress IFN-b, a key factor in thymic events leading to MG [3].

The temporal association of neuromuscular disorders and COVID-19 vaccination cannot easily be translated into a causal relationship, and the evidence in this regard is inadequate [151]. Thus, further studies are required to clarify this association.

SFN is categorized as primary and secondary to vaccination or diseases such as kidney failure, diabetes, infections, or autoimmune. As Finsterer et al.129 noted, the clinical presentation of COVID-19 vaccine-associated SFN is similar to other secondary SFNs. Also, evidence on the beneficial effects of IVIG supports the autoimmune nature of the SFN associated with COVID-19 vaccines.

The median time from vaccine to symptom was less than 2 weeks in all NMD, mainly after the first dose; a case can be made that vaccination may have exacerbated an already existing but asymptomatic form in the vaccinated individuals instead of triggering a new onset of the disease.

### Limitation and strengths

This review acknowledges certain limitations inherent in our study design. Firstly, due to the nature of the collected data, We couldn’t analyze the link between the number of COVID-19 vaccine doses and neuromuscular disorders. Furthermore, we couldn’t assess the effects of mixing different COVID-19 vaccines for primary and booster doses. Our study can’t conclusively establish a causal relationship between COVID-19 vaccination and neuromuscular events. Few cases of neuromuscular disorders like Tolosa-Hunt syndrome and SFN were reported.

Furthermore, our pooled data sets were incomplete for some variables, such as laboratory tests or imaging. However, we pooled data from all reported cases of NMD following COVID-19 vaccines using a comprehensive search strategy to provide a better understanding of this issue. To our knowledge, this is the first systematic review of reported cases of COVID-19 vaccine-associated NMD.

## Conclusion

Based on our comprehensive literature review, COVID-19 vaccines might induce some neuromuscular diseases. These cases mainly occurred after administering the most frequently used COVID-19 vaccines. The age distribution and gender characteristics of affected patients may differ based on the NMD type. About two-thirds of the cases occurred in less than 2 weeks after vaccination. Most cases developed the symptoms following the first dose of vector-based vaccines. The majority of these patients experienced a benign disease course.

### Supplementary Information

**Additional file 1: Supplementary Table 1. **Quality assessment of the included case series based on the JBI checklist for case series. **Supplementary Table 2.** Guillain barre syndrome studies and patients’ characteristics by case. **Supplementary Table 3.** Guillain Barre patients’ findings, treatments, and outcomes by case. **Supplementary Table 4.** Parsonage turner studies and patients’ characteristics by case. **Supplementary Table 5.** Parsonage turner patients’ findings, treatments, and outcomes by case. **Supplementary table 6.** Facial nerve palsy patients’ findings, treatments, and clinical outcomes by case. **Supplementary table 7.**  Small fiber neuropathy and tolosa-hunt patients’ findings, treatments, and clinical outcomes by case. **Supplementary Table 8.** Myasthenia gravis studies and patients’ characteristics by case. **Supplementary Table 9.** Myasthenia gravis patients’ findings, treatments, and clinical outcomes by case.

## Data Availability

All data and materials used in this study are accessible upon request. For inquiries regarding data and materials, please contact the corresponding author.
