# Supplementary material for: Neuromuscular diseases associated with COVID-19 vaccines: a systematic review and pooled analysis of 258 patients
Source: BMC Neurol. 2023 Dec 11;23:437. doi: 10.1186/s12883-023-03486-y (PMC10712145; doi:10.1186/s12883-023-03486-y)
Supplement: Supplementary file 1 — Additional file 1: Supplementary Table 1. Quality assessment of the included case series based on the JBI checklist for case series. Supplementary Table 2. Guillain barre syndrome studies and patients’ characteristics by case. Supplementary Table 3. Guillain Barre patients’ findings, treatments, and outcomes by case. Supplementary Table 4. Parsonage turner studies and patients’ characteristics by case. Supplementary Table 5. Parsonage turner patients’ findings, treatments, and outcomes by case. Supplementary table 6. Facial nerve palsy patients’ findings, treatments, and clinical outcomes by case. Supplementary table 7. Small fiber neuropathy and tolosa-hunt patients’ findings, treatments, and clinical outcomes by case. Supplementary Table 8. Myasthenia gravis studies and patients’ characteristics by case. Supplementary Table 9. Myasthenia gravis patients’ findings, treatments, and clinical outcomes by case. [file 12883_2023_3486_MOESM1_ESM.docx]

| **Supplementary Table 1:** Quality assessment of the included case series based on the JBI checklist for case series. | | | | | | | | | | | | | | | | | | | | | | | | | | | | | | | |
| --- | --- | --- | --- | --- | --- | --- | --- | --- | --- | --- | --- | --- | --- | --- | --- | --- | --- | --- | --- | --- | --- | --- | --- | --- | --- | --- | --- | --- | --- | --- | --- |
| **Items** | Allen et al. | Bax et al. | Bonifacio et al. et al. | Garcia et al. | James et al. | Kanabar et al. | Karimi et al. | Maramottom et al. | Min et al. | Oo et al. | Osowicki et al. | Castiglione et al. | Kim et al. | Chun et al. | Germano et al. | Hai et al, | Berrim et al. | J.Kim et al. | Nagadev et al. | Tabatabaee rt al. | Wan et al. | Shields et al. | Queler et al. | Min et al. | Koh et al. | Mirmosayyeb et al. | Salbas et al. | Finstere et al. | Fanella et al. | Ramdas et al. | Wated et al. |
| 1 | + | + | + | + | + | + | + | + | + | + | + | + | + | + | + | + | + | + | + | + | + | + | + | + | + | + | + | + | + | + | + |
| 2 | + | + | + | + | + | + | + | + | + | + | + | + | + | + | + | + | + | + | + | + | + | + | + | + | + | + | + | + | + | + | + |
| 3 | + | + | + | + | + | + | + | + | + | + | + | + | + | + | + | + | + | + | + | + | + | + | + | + | + | + | + | + | + | + | + |
| 4 | - | - | + | + | ­- | - | + | + | - | - | - | + | + | - | + | - | + | - | - | - | - | - | + | + | - | - | - | - | + | + | + |
| 5 | - | - | + | + | - | - | + | + | - | - | - | + | + | - | + | - | + | - | - | - | - | - | + | + | - | - | - | - | + | + | - |
| 6 | + | + | + | + | + | + | + | + | + | + | + | + | + | + | + | + | + | + | + | + | + | + | + | + | + | + | + | + | + | + | + |
| 7 | + | + | + | + | + | + | + | + | + | + | + | + | + | + | + | + | + | + | + | + | + | + | + | + | + | + | + | + | + | - | - |
| 8 | + | + | + | + | + | + | - | - | - | - | - | - | - | - | + | - | + | + | - | - | + | + | + | + | + | + | + | - | + | - | - |
| 9 | + | + | + | + | + | + | + | + | + | + | + | + | + | + | + | + | + | + | + | + | + | + | + | + | + | + | + | + | + | - | + |
| 10 | + | + | + | + | + | + | + | + | + | + | + | + | + | + | + | + | + | + | + | + | + | + | + | + | + | + | + | + | + | + | + |
| Total score | 8 | 8 | 10 | 10 | 8 | 8 | 9 | 9 | 7 | 7 | 7 | 9 | 9 | 7 | 10 | 7 | 10 | 8 | 7 | 7 | 8 | 8 | 10 | 10 | 8 | 8 | 8 | 7 | 10 | 7 | 7 |
| 1. Were there clear criteria for inclusion in the case series?  2. Was the condition measured in a standard, reliable way for all participants included in the case series?  3. Were valid methods used for identification of the condition for all participants included in the case series?  4. Did the case series have consecutive inclusion of participants?  5. Did the case series have complete inclusion of participants?  6. Was there clear reporting of the demographics of the participants in the study?  7. Was there clear reporting of clinical information of the participants?  8. Were the outcomes or follow-up results of cases clearly reported?  9. Was there clear reporting of the presenting site(s)/clinic(s) demographic information?  10. Was statistical analysis appropriate? | | | | | | | | | | | | | | | | | | | | | | | | | | | | | | | |

| **Supplementary Table 2:** Guillain barre syndrome studies and patients’ characteristics by case. | | | | | | | | | | |
| --- | --- | --- | --- | --- | --- | --- | --- | --- | --- | --- |
| **#** | **Author** | **Year** | **Country** | **Sex** | **Age** | **Vaccine type** | **Vaccine dose** | **Time to onset** | **GBS type** | **EMG/NCV** |
| 1 | Abicic et al. | 2021 | Croatia | F | 24 | Pfizer | 1^st^ dose | 18 | MFS | NR |
| 2 | Allen et al. | 2021 | UK | M | 54 | AstraZeneca | 1^st^ dose | 16 | BFP | NR |
| 3 |  |  |  | M | 20 | AstraZeneca | 1^st^ dose | 26 | BFP | NR |
| 4 |  |  |  | M | 57 | AstraZeneca | 1^st^ dose | 21 | BFP | - |
| 5 |  |  |  | M | 55 | AstraZeneca | 1^st^ dose | 29 | BFP | NR |
| 6 | Aomar-millan et al. | 2021 | Spain | M | 77 | Pfizer | 1^st^ dose | 3 | AMSAN | + |
| 7 | Azam et al. | 2021 | UK | M | 67 | AstraZeneca | 1^st^ dose | 15 | AIDP | + |
| 8 | Bax et al. | 2021 | Italy | m | 90 | Pfizer | 2^nd^ dose | 3 | AMSAN | + |
| 9 |  |  |  | F | 51 | AstraZeneca | 1^st^ dose | 10 | AIDP | + |
| 10 | Bonifacio et al. | 2022 | UK | M | 66 | AstraZeneca | 1^st^ dose | 17 | BFP | + |
| 11 |  |  |  | M | 43 | AstraZeneca | 1^st^ dose | 17 | BFP | + |
| 12 |  |  |  | M | 51 | AstraZeneca | 1^st^ dose | 14 | BFP | + |
| 13 |  |  |  | F | 71 | AstraZeneca | 1^st^ dose | 15 | BFP | + |
| 14 |  |  |  | M | 33 | AstraZeneca | 1^st^ dose | 14 | BFP | NR |
| 15 | Bouattour et al. | 2021 | Tunisia | M | 67 | Pfizer | 1^st^ dose | 7 | AIDP | + |
| 16 | Dang et al. | 2021 | Australia | M | 63 | AstraZeneca | 1^st^ dose | 14 | MFS + Classic GBS | NR |
| 17 | Da Silva et al. | 2022 | Brazil | F | 62 | AstraZeneca | 1^st^ dose | 18 | Classic SM | NR |
| 18 | Finsterer et al. | 2021 | Austria | M | 32 | NR | 1^st^ dose | 8 | AIDP | + |
| 19 | Garcia et al. | 2021 | Mexico | M | 33 | Pfizer | 1^st^ dose | 28 | AIDP | + |
| 20 |  |  |  | M | 25 | Pfizer | 1^st^ dose | 12 | AIDP | + |
| 21 |  |  |  | F | 53 | Pfizer | 1^st^ dose | 6 | AMAN | + |
| 22 |  |  |  | M | 72 | Pfizer | 1^st^ dose | 4 | AMAN | + |
| 23 |  |  |  | M | 31 | Pfizer | 1^st^ dose | 11 | AIDP | + |
| 24 |  |  |  | F | 67 | Pfizer | 1^st^ dose | 4 | AMAN | + |
| 25 |  |  |  | F | 81 | Pfizer | 1^st^ dose | 3 | AIDP | + |
| 26 | Hasan et al. | 2021 | UK | F | 62 | AstraZeneca | 1^st^ dose | 11 | AIDP | + |
| 27 | Hughes et al. | 2022 | USA | M | 65 | Pfizer | 1^st^ dose | 2 | AIDP | + |
| 28 | Introna et al. | 2021 | Italy | M | 62 | AstraZeneca | 1^st^ dose | 10 | AIDP | + |
| 29 | Jain et al. | 2021 | USA | F | 65 | J&J | NR | 19 | BFP | NR |
| 30 | James et al. | 2021 | India | M | 60 | AstraZeneca | 1^st^ dose | 11 | AMSAN | + |
| 31 |  |  |  | M | 66 | AstraZeneca | 1^st^ dose | 12 | AIDP | + |
| 32 |  |  |  | F | 54 | AstraZeneca | 1^st^ dose | 13 | AIDP | + |
| 33 | Kanabar et al. | 2021 | UK | F | 61 | AstraZeneca | 1^st^ dose | 10 | AIDP | + |
| 34 |  |  |  | M | 56 | AstraZeneca | 1^st^ dose | NR | AIDP | + |
| 35 | Karimi et al. | 2021 | Iran | M | 38 | Sputnik | NR | 14 | BFP | + |
| 36 |  |  |  | M | 38 | Sputnik | 1^st^ dose | 8 | AIDP | + |
| 37 |  |  |  | M | 87 | Sinopharm | 1^st^ dose | 4 | AIDP | + |
| 38 |  |  |  | M | 52 | Sputnik | NR | 21 | AIDP | + |
| 39 |  |  |  | F | 48 | Sputnik | NR | 17 | Classic SM | NR |
| 40 |  |  |  | F | 26 | Sinopharm | 2^nd^ dose | 37 | AIDP | + |
| 41 |  |  |  | M | 44 | AstraZeneca | 1^st^ dose | 14 | AMSAN | + |
| 42 |  |  |  | M | 76 | Sinopharm | 1^st^ dose | 14 | AMAN | + |
| 43 |  |  |  | M | 79 | AstraZeneca | 1^st^ dose | 7 | AMSAN | + |
| 44 | Ling et al. | 2021 | Canada | M | 63 | AstraZeneca | 1^st^ dose | 12 | AIDP | + |
| 45 | Loza et al. | 2021 | USA | F | 60 | J&J | NR | 10 | MFS + Classic GBS | + |
| 46 | Malamud et al. | 2022 | USA | M | 14 | Pfizer | 2^nd^ dose | 30 | AIDP | + |
| 47 | Maramottom et al. | 2021 | India | F | 43 | AstraZeneca | 1^st^ dose | 10 | AIDP | + |
| 48 |  |  |  | F | 67 | AstraZeneca | 1^st^ dose | 14 | AMSAN | + |
| 49 |  |  |  | F | 53 | AstraZeneca | 1^st^ dose | 12 | AIDP | + |
| 50 |  |  |  | F | 68 | AstraZeneca | 1^st^ dose | 14 | AIDP | + |
| 51 |  |  |  | M | 70 | AstraZeneca | 1^st^ dose | 11 | AIDP | + |
| 52 |  |  |  | F | 69 | AstraZeneca | 1^st^ dose | 12 | AIDP | + |
| 53 |  |  |  | F | 69 | AstraZeneca | 1^st^ dose | 13 | AIDP | + |
| 54 | Masuccio et al. | 2021 | Italy | M | 80 | Moderna | 2^nd^ dose | 44 | AIDP | + |
| 55 | Matarneh et al. | 2021 | Qatar | M | 61 | Moderna | 2^nd^ dose | 4 | Pure motor | + |
| 56 | McKean et al. | 2021 | Malta | M | 48 | AstraZeneca | 1^st^ dose | 10 | AIDP | + |
| 57 | Michaelson et al. | 2021 | USA | M | 78 | Pfizer | 2^nd^ dose | 14 | MFS | + |
| 58 | Min et al. | 2021 | South Korea | M | 58 | AstraZeneca | 1^st^ dose | 15 | Sensory | NR |
| 59 |  |  |  | F | 73 | AstraZeneca | 1^st^ dose | 18 | Sensory | - |
| 60 | Morehouse et al. | 2021 | USA | F | 49 | J&J | NR | 5 | Classic SM | NR |
| 61 | Nasuelli et al. | 2021 | Italy | M | 59 | AstraZeneca | 1^st^ dose | 10 | AIDP | + |
| 62 | Nishiguchi et al. | 2021 | Japan | M | 71 | Pfizer | 1^st^ dose | 18 | MFS | + |
| 63 | Ogbebor et al. | 2021 | USA | F | 86 | Pfizer | 1^st^ dose | 1 | Paraparetic | NR |
| 64 | Oo et al. | 2021 | Australia | M | 51 | AstraZeneca | 1^st^ dose | 14 | AIDP | + |
| 65 |  |  |  | F | 65 | AstraZeneca | 1^st^ dose | 7 | AIDP | + |
| 66 |  |  |  | M | 66 | AstraZeneca | 1^st^ dose | 21 | AIDP | + |
| 67 | Osowicki et al. | 2021 | Australia | F | 75 | AstraZeneca | 1^st^ dose | 17 | AIDP | + |
| 68 |  |  |  | F | 77 | AstraZeneca | 1^st^ dose | 17 | AIDP | + |
| 69 |  |  |  | F | 57 | AstraZeneca | 1^st^ dose | 13 | AIDP | + |
| 70 |  |  |  | M | 57 | AstraZeneca | 1^st^ dose | 12 | Paraparetic | NR |
| 71 |  |  |  | F | 52 | AstraZeneca | 1^st^ dose | 20 | BFP | NR |
| 72 |  |  |  | M | 54 | AstraZeneca | 1^st^ dose | 10 | AIDP | + |
| 73 |  |  |  | F | 80 | AstraZeneca | 1^st^ dose | 21 | Paraparetic | NR |
| 74 |  |  |  | M | 72 | AstraZeneca | 1^st^ dose | 14 | Classic | NR |
| 75 |  |  |  | M | 59 | AstraZeneca | 1^st^ dose | 25 | Classic | NR |
| 76 |  |  |  | M | 69 | AstraZeneca | 1^st^ dose | 16 | Classic | NR |
| 77 |  |  |  | F | 72 | AstraZeneca | 1^st^ dose | 11 | Classic | NR |
| 78 |  |  |  | M | 66 | AstraZeneca | 1^st^ dose | 11 | Classic | NR |
| 79 |  |  |  | M | 63 | AstraZeneca | 1^st^ dose | 14 | Classic | NR |
| 80 |  |  |  | M | 70 | AstraZeneca | 1^st^ dose | 14 | AMSAN | + |
| 81 | Patel et al. | 2021 | UK | M | 37 | AstraZeneca | 1^st^ dose | 14 | Classic SM | + |
| 82 | Prasad et al. | 2021 | USA | M | 41 | J&J | NR | 15 | BFP | + |
| 83 | Razok et al. | 2021 | Qatar | M | 73 | Pfizer | 2^nd^ dose | 16 | Paraparetic | + |
| 84 | Rossetti et al. | 2021 | USA | N | 38 | J&J | NR | 30 | BFP | NR |
| 85 | Scendoni et al. | 2021 | Italy | F | 82 | Pfizer | 2^nd^ dose | 14 | AIDP | + |
| 86 | Trimboli et al. | 2021 | Italy | F | 25 | Pfizer | 2^nd^ dose | 8 | AIDP | + |
| 87 | Tutar et al. | 2021 | Turkey | M | 76 | CoronaVac | 2^nd^ dose | 8 | AMSAN | + |
| 88 | Waheed et al. | 2021 | USA | F | 83 | Pfizer | 1^st^ dose | 14 | Classic SM | NR |
| 89 | Anjum et al. | 2022 | Nepal | M | 80 | Moderna | 2^nd^ dose | 1 | AIDP | + |
| 90 | Bazrafshan et al. | 2022 | Iran | F | 68 | AstraZeneca | 2^nd^ dose | 3 | AIDP | + |
| 91 | Bellucci et al. | 2022 | Japan | M | 57 | Pfizer | 1^st^ dose | 5 | AIDP | + |
| 92 | Castiglione et al. | 2022 | Argentina | F | 56 | Sputnik | 1^st^ dose | 19 | FDFN | + |
| 93 |  |  |  | M | 55 | Sputnik | 1^st^ dose | 28 | FDFN | + |
| 94 |  |  |  | M | 87 | Sputnik | 1^st^ dose | 17 | AIDP | + |
| 95 |  |  |  | M | 50 | AstraZeneca | 1^st^ dose | 20 | AIDP | + |
| 96 |  |  |  | M | 39 | Sputnik | 1^st^ dose | 10 | FDFN | + |
| 97 |  |  |  | M | 42 | AstraZeneca | 1^st^ dose | 28 | FDFN | + |
| 98 |  |  |  | F | 52 | AstraZeneca | 1^st^ dose | 13 | AIDP | + |
| 99 |  |  |  | M | 43 | Sputnik | 2^nd^ dose | 13 | AIDP | + |
| 100 |  |  |  | M | 65 | AstraZeneca | 2^nd^ dose | 13 | FDFN | + |
| 101 | Kim et al. | 2021 | South Korea | M | 62 | AstraZeneca | 1^st^ dose | 11 | Classic | + |
| 102 |  |  |  | F | 73 | AstraZeneca | 1^st^ dose | 29 | Classic | + |
| 103 |  |  |  | F | 48 | AstraZeneca | 1^st^ dose | 10 | Paraparetic | + |
| 104 |  |  |  | F | 32 | Pfizer | 1^st^ dose | 11 | NR | + |
| 105 |  |  |  | M | 38 | AstraZeneca | 1^st^ dose | 29 | Classic | + |
| 106 |  |  |  | F | 70 | AstraZeneca | 1^st^ dose | 8 | Classic | + |
| 107 |  |  |  | M | 72 | AstraZeneca | 1^st^ dose | 30 | Classic | NR |
| 108 |  |  |  | F | 84 | AstraZeneca | 1^st^ dose | 8 | Classic | - |
| 109 |  |  |  | F | 62 | AstraZeneca | 2^nd^ dose | 27 | Classic | NR |
| 110 |  |  |  | M | 43 | Pfizer | 1^st^ dose | 11 | Classic | + |
| 111 |  |  |  | F | 18 | Pfizer | 1^st^ dose | 12 | Paraparetic | - |
| 112 |  |  |  | F | 73 | Pfizer | 1^st^ dose | 13 | Paraparetic | NR |
| 113 |  |  |  | F | 58 | Pfizer | 1^st^ dose | 4 | Classic | + |
| 114 | Censcak et al. | 2021 | Czech | M | 42 | Pfizer | 1^st^ dose | 14 | AIDP | + |
| 115 | Chun et al. | 2022 | South Korea | F | 80 | Pfizer | 2^nd^ dose | 8 | NR | NR |
| 116 |  |  |  | F | 76 | Pfizer | 2^nd^ dose | 22 | AIDP | + |
| 117 | Donaldson et al. | 2022 | Canada | M | 45 | AstraZeneca | 1^st^ dose | 12 | NR | + |
| 118 | Germano et al. | 2022 | Italy | M | 68 | AstraZeneca | 1^st^ dose | 12 | AIDP | + |
| 119 |  |  |  | F | 71 | Pfizer | 1^st^ dose | 10 | AMSAN | + |
| 120 |  |  |  | F | 40 | Pfizer | 2^nd^ dose | 4 | AIDP | + |
| 121 |  |  |  | M | 89 | Moderna | 1^st^ dose | 15 | AMSAN | + |
| 122 |  |  |  | M | 65 | AstraZeneca | 1^st^ dose | 15 | AIDP | + |
| 123 |  |  |  | M | 80 | Pfizer | 2^nd^ dose | 21 | AIDP | + |
| 124 |  |  |  | F | 69 | AstraZeneca | 1^st^ dose | 17 | AIDP | + |
| 125 |  |  |  | M | 18 | Pfizer | 1^st^ dose | 12 | AIDP | + |
| 126 |  |  |  | M | 57 | Pfizer | 1^st^ dose | 5 | AIDP | + |
| 127 |  |  |  | M | 64 | AstraZeneca | 1^st^ dose | 15 | AIDP | + |
| 128 |  |  |  | M | 88 | Pfizer | 2^nd^ dose | 15 | AIDP | + |
| 129 |  |  |  | F | 73 | AstraZeneca | 1^st^ dose | 5 | AIDP | + |
| 130 |  |  |  | M | 51 | Pfizer | 1^st^ dose | 4 | AIDP | + |
| 131 | Gunawan et al. | 2022 | Indonesia | M | 14 | CoronaVac | 2^nd^ dose | 21 | AIDP | + |
| 132 | Hai et al. | 2022 | Vietnam | M | 38 | AstraZeneca | 1^st^ dose | 4 | NR | + |
| 133 |  |  |  | M | 29 | AstraZeneca | 2^nd^ dose | 21 | NR | NR |
| 134 | Hilts et al. | 2022 | USA | M | 58 | Moderna | 1^st^ dose | 3 | NR | NR |
| 135 | Liang et al. | 2022 | China | M | 64 | Sinovac-CoronaVac | 2^nd^ dose | 10 | MFS | NR |
| 136 | Richardson-May et al. | 2022 | The UK | F | 71 | Vaxzevria | 1^st^ dose | 14 | NR | + |
| 137 | Prado et al. | 2022 | Philippine | M | 35 | NR | 2^nd^ dose | 2 | NR | + |
| 138 | Berrim et al. | 2022 | Tunisia | F | 41 | Vaxzevria | 2^nd^ dose | 7 | AIDP | + |
| 139 |  |  |  | M | 53 | Vaxzevria | 1^st^ dose | 7 | AIDP | + |
| 140 |  |  |  | M | 80 | J&J | 1^st^ dose | 30 | AIDP | + |
| 141 |  |  |  | M | 62 | Pfizer | Booster | 28 | AIDP | + |
| 142 | Finsterer et al. | 2021 | Austria | F | 69 | NR | 1^st^ dose | 40 | AIDP | + |
| 143 | Khadka et al. | 2021 | Nepal | M | 44 | J&J | NR | 15 | AIDP | + |
| 144 | Hwang et al. | 2022 | South Korea | M | 47 | Moderna | 1^st^ dose | 1 | AIDP | + |
| 145 | Ilyas et al. | 2022 | USA | M | 62 | Pfizer | Booster | 6 | AIDP | NR |
| 146 | Y.Kim et al. | 2021 | USA | F | 16 | Pfizer | 2^nd^ dose | 2 | AIDP | + |
| 147 | N. Kim et al. | 2022 | South Korea | M | 21 | Pfizer | 1^st^ dose | 21 | BFP | NR |
| 148 | J. Kim et al. | 2021 | South Korea | M | 42 | AstraZeneca | 1^st^ dose | 14 | BFP | + |
| 149 |  |  |  | F | 48 | Pfizer | 1^st^ dose | 14 | Sensory motor | + |
| 150 | Kripalani et al. | 2021 | India | F | 52 | AstraZeneca | 1^st^ dose | 9 | AIDP | + |
| 151 | Lanman et al. | 2022 | USA | F | 58 | Pfizer | 1^st^ dose | 3 | AMSAN | + |
| 152 | Nagalli et al. | 2021 | USA | F | 49 | Moderna | 1^st^ dose | 10 | AIDP | + |
| 153 | Nagdev et al. | 2022 | India | M | 40 | AstraZeneca | 1^st^ dose | 12 | AIDP | + |
| 154 |  |  |  | F | 23 | AstraZeneca | NR | 15 | AIDP | + |
| 155 |  |  |  | M | 20 | AstraZeneca | NR | 14 | Sensory motor | + |
| 156 |  |  |  | M | 25 | AstraZeneca | 1^st^ dose | 15 | Sensory motor | + |
| 157 |  |  |  | M | 62 | AstraZeneca | 1^st^ dose | 10 | Sensory motor | + |
| 158 | Nanatsue et al. | 2022 | Japan | M | 70 | Moderna | 2^nd^ dose | 7 | MFS | NR |
| 159 | Ogata et al. | 2022 | Japan | M | 70 | Pfizer | 1^st^ dose | 23 | AIDP | + |
| 160 | Pirola et al. | 2022 | Brazil | F | 47 | AstraZeneca | 1^st^ dose | 3 | MFS | + |
| 161 | Rao et al. | 2021 | USA | F | 42 | Pfizer | 2^nd^ dose | 7 | NR | + |
| 162 | Siddiqi et al. | 2022 | Pakistan | M | 58 | Sinovac | 1^st^ dose | 8 | MFS | NR |
| 163 | Thant et al. | 2022 | USA | M | 66 | J&J | 1^st^ dose | 14 | AIDP | NR |
| 164 | Theuriet et al. | 2021 | Switzerland | M | 72 | AstraZeneca | 1^st^ dose | 21 | AIDP | + |
| 165 | Zubair et al. | 2022 | USA | F | 30 | J&J | NR | 14 | NR | NR |
| 166 | Tabatabaee et al. | 2022 | Iran | M | 46 | AstraZeneca | 2^nd^ dose | 3 | AMAN | + |
| 167 |  |  |  | M | 36 | Sinopharm | 1^st^ dose | 5 | AMAN | + |
| 168 |  |  |  | M | 32 | Sinopharm | 1^st^ dose | 14 | AMAN | + |
| 169 | Wan et al. | 2022 | Canada | M | 40 | AstraZeneca | 1^st^ dose | 10 | AIDP | + |
| 170 |  |  |  | F | 53 | AstraZeneca | 1^st^ dose | 12 | Classic GBS | + |
| 171 |  |  |  | M | 59 | AstraZeneca | 1^st^ dose | 14 | NR | + |
| **AIDP:** Acute inflammatory demyelinating polyneuropathy, **AMAN:** Acute motor axonal neuropathy, **AMSAN:** Acute motor and sensory axonal neuropathy, **BFP:** Bilateral facial palsy, **EMG:** Electromyography, **F:** Female, **FDFN:** Focal demyelination of facial nerves, **J&J:** Johnson and Johnsons, **M:** Male, **MFS:** Miller fisher syndrome, **NCV:** Nerve conduction velocity, **NR:** Not reported, **SM:** Sensory motor | | | | | | | | | | |

| **Supplementary Table 3:** Guillain Barre patients’ findings, treatments, and outcomes by case. | | | | | | |
| --- | --- | --- | --- | --- | --- | --- |
| **#** | **Albuminocytological dissociation** | **Anti ganglioside Antibodies** | **MRI** | **Treatment** | **Intubation** | **Outcomes** |
| 1 | NR | + | NR | Corticosteroids IVIG | - | Complete recovery |
| 2 | + | - | NR | Corticosteroids | - | Complete recovery |
| 3 | + | - | NL | Corticosteroids | - | Complete recovery |
| 4 | + | - | NL | IVIG | - | Complete recovery |
| 5 | + | NR | Right facial nerve enhancement | Conservative | - | Complete recovery |
| 6 | - | - | NR | IVIG  PLEX | - | Complete recovery |
| 7 | + | - | Bilateral facial nerve enhancement | IVIG | - | NR |
| 8 | + | NR | NR | IVIG | - | Complete recovery |
| 9 | NR | - | NR | IVIG | - | Complete recovery |
| 10 | + | - | NR | IVIG | NR | Complete recovery |
| 11 | + | - | NR | IVIG | NR | Partial recovery |
| 12 | + | Anti GM3 + | NR | NR | NR | Complete recovery |
| 13 | + | - | NL | NR | NR | Complete recovery |
| 14 | + | NR | NR | NR | NR | Complete recovery |
| 15 | + | - | NR | IVIG | - | Complete recovery |
| 16 | + | - | Bilateral optic and oculomotor enhancement | IVIG | - | Partial recovery |
| 17 | + | NR | NR | IVIG | - | Complete recovery |
| 18 | + | NR | Non-specific bilateral white matter hyperintensity | IVIG | - | Partial recovery |
| 19 | + | NR | NR | IVIG | - | Complete recovery |
| 20 | + | NR | NR | IVIG | - | Partial recovery |
| 21 | - | NR | NR | IVIG | + | Poor recovery |
| 22 | NR | NR | NR | IVIG | - | Partial recovery |
| 23 | NR | NR | NR | IVIG | - | Partial recovery |
| 24 | - | NR | NR | IVIG | + | Died |
| 25 | + | NR | NR | IVIG | - | Partial recovery |
| 26 | + | NR | NL | IVIG | + | Poor recovery |
| 27 | + | NR | NL | IVIG | - | Complete recovery |
| 28 | + | Anti GM1 + | NL | IVIG | - | Partial recovery |
| 29 | + | - | NL | IVIG  PLEX | - | Complete recovery |
| 30 | + | NR | NL | IVIG | - | Complete recovery |
| 31 | + | NR | NL | IVIG  Corticosteroids | - | Complete recovery |
| 32 | NR | - | NL | IVIG  Corticosteroids | - | Complete recovery |
| 33 | + | NR | NR | IVIG | - | Complete recovery |
| 34 | + | NR | NR | IVIG | - | Complete recovery |
| 35 | + | NR | NL | PLEX | - | Complete recovery |
| 36 | + | NR | NL | PLEX | - | Partial recovery |
| 37 | + | NR | NL | IVIG | - | Partial recovery |
| 38 | + | NR | NL | IVIG | - | Complete recovery |
| 39 | NR | NR | NR | IVIG  PLEX | + | Partial recovery |
| 40 | - | NR | NR | IVIG | - | Complete recovery |
| 41 | + | NR | NR | IVIG | - | Complete recovery |
| 42 | + | NR | NR | PLEX | - | Complete recovery |
| 43 | NR | NR | NR | PLEX | - | Partial recovery |
| 44 | + | NR | NL | IVIG  PLEX | - | Partial recovery |
| 45 | + | - | Cauda equina enhancement | IVIG | - | Complete recovery |
| 46 | + | NR | NR | IVIG | - | Complete recovery |
| 47 | + | NR | NR | IVIG | + | Complete recovery |
| 48 | + | - | NL | IVIG  PLEX | + | Poor recovery |
| 49 | + | - | NL | IVIG | + | Poor recovery |
| 50 | + | - | NL | IVIG | + | Poor recovery |
| 51 | NR | NR | NR | IVIG | + | Poor recovery |
| 52 | NR | NR | NR | IVIG  PLEX | - | Poor recovery |
| 53 | + | NR | NR | IVIG | + | Poor recovery |
| 54 | + | - | NL | IVIG | - | Complete recovery |
| 55 | + | NR | NR | IVIG | - | Complete recovery |
| 56 | + | - | NL | IVIG  Corticosteroids | - | Complete recovery |
| 57 | + | - | NL | IVIG | - | Complete recovery |
| 58 | + | - | NL | Gabapentin | - | Complete recovery |
| 59 | NR | NR | NR | Gabapentin  Duloxetine  Tramadol | NR | Complete recovery |
| 60 | - | NR | Small punctate foci | IVIG  PLEX | + | Poor recovery |
| 61 | + | - | NL | IVIG | - | Complete recovery |
| 62 | + | - | NL | IVIG | - | Complete recovery |
| 63 | + | NR | NL | IVIG | - | Complete recovery |
| 64 | + | NR | NR | IVIG  PLEX | + | Poor recovery |
| 65 | + | - | NL | IVIG | + | Partial recovery |
| 66 | + | NR | NR | IVIG | - | Complete recovery |
| 67 | NR | NR | NR | NR | NR | NR |
| 68 | NR | NR | NR | NR | NR | NR |
| 69 | NR | NR | NR | NR | NR | NR |
| 70 | NR | NR | NR | NR | NR | NR |
| 71 | NR | NR | NR | NR | NR | NR |
| 72 | NR | NR | NR | NR | NR | NR |
| 73 | NR | NR | NR | NR | NR | NR |
| 74 | NR | NR | NR | NR | NR | NR |
| 75 | NR | NR | NR | NR | NR | NR |
| 76 | NR | NR | NR | NR | NR | NR |
| 77 | NR | NR | NR | NR | NR | NR |
| 78 | NR | NR | NR | NR | NR | NR |
| 79 | NR | NR | NR | NR | NR | NR |
| 80 | NR | NR | NR | NR | NR | NR |
| 81 | + | NR | Prominent ventral Cauda equina nerve root enhancement | IVIG | - | Complete recovery |
| 82 | + | NR | NL | IVIG | - | Partial recovery |
| 83 | + | NR | Bilateral lumbar nerve root enhancement | IVIG | - | Complete recovery |
| 84 | + | NR | Focal bilateral enhancement of the internal auditory canal, fundi and cisternal segments of the trigeminal nerves | IVIG | - | Complete recovery |
| 85 | + | + | NR | IVIG | - | Partial recovery |
| 86 | - | NR | NR | IVIG | - | Complete recovery |
| 87 | - | ­- | NL | IVIG | - | Complete recovery |
| 88 | + | NR | Cauda equina nerve root enhancement | IVIG | - | Partial recovery |
| 89 | + | NR | Consistent with degenerative disease | IVIG | - | Partial recovery |
| 90 | + | NR | NL | IVIG | - | Complete recovery |
| 91 | + | Anti GM3,4 +  Anti GD1a, b +  Anti GT1b IgM + | NR | IVIG | - | Complete recovery |
| 92 | - | - | NR | IVIG | - | Partial recovery |
| 93 | + | - | NR | IVIG | - | Partial recovery |
| 94 | + | Anti GD1a + | NR | NR | - | Died |
| 95 | + | - | NR | IVIG | - | Partial recovery |
| 96 | - | + | NR | IVIG | - | Partial recovery |
| 97 | + | NR | NR | IVIG | - | Partial recovery |
| 98 | + | Anti GM1 + | NR | PLEX | - | Partial recovery |
| 99 | + | - | NR | IVIG | - | Partial recovery |
| 100 | + | Anti GM1 + | NR | IVIG | - | Partial recovery |
| 101 | NR | - | NR | IVIG | - | NR |
| 102 | NR | - | NR | IVIG | - | NR |
| 103 | NR | - | NR | Corticosteroids | - | NR |
| 104 | NR | - | NR | IVIG | - | NR |
| 105 | NR | - | NR | NR | - | NR |
| 106 | NR | Anti GM1 IgM + | NR | IVIG | - | NR |
| 107 | NR | NR | NR | NR | - | NR |
| 108 | NR | Anti GQ1b IgG + | NR | NR | - | NR |
| 109 | NR | - | NR | IVIG | - | NR |
| 110 | NR | - | NR | IVIG | - | NR |
| 111 | NR | - | NR | NR | - | NR |
| 112 | NR | - | NR | IVIG | - | NR |
| 113 | NR | - | NR | IVIG | - | NR |
| 114 | + | - | mild thickening and enhancement of spinal roots in lumbosacral region. | IVIG | - | Partial recovery |
| 115 | NR | NR | NR | Corticosteroids  IVIG | - | NR |
| 116 | + | - | NR | IVIG | - | NR |
| 117 | + | - | NL | IVIG | - | Complete recovery |
| 118 | + | NR | NR | IVIG | - | NR |
| 119 | + | NR | NR | IVIG | - | NR |
| 120 | + | NR | NR | IVIG | - | NR |
| 121 | + | NR | NR | IVIG | - | NR |
| 122 | + | NR | NR | PLEX | - | NR |
| 123 | + | NR | NR | IVIG | - | NR |
| 124 | - | NR | NR | IVIG | - | NR |
| 125 | - | NR | NR | IVIG | - | NR |
| 126 | + | NR | NR | IVIG | - | NR |
| 127 | NR | NR | NR | IVIG | - | NR |
| 128 | + | NR | NR | IVIG | - | NR |
| 129 | - | NR | NR | NR | - | NR |
| 130 | - | NR | NR | NR | - | NR |
| 131 | NR | NR | NR | NR | - | Partial recovery |
| 132 | + | NR | NR | PLEX | - | NR |
| 133 | + | NR | NR | PLEX | - | NR |
| 134 | ­+ | NR | Degenerative changes with some stenosis, most significant at C5/6 | IVIG  PLEX | - | Partial recovery |
| 135 | + | anti-GQ1b IgG +  anti-GT1b IgG + | NL | IVIG | - | Complete recovery |
| 136 | + | + | Acute infarct  Carotid artery duplex showed intimal thickening | Prednisolone | - | Partial recovery |
| 137 | + | - | NR | Acyclovir  Corticosteroids | - | NR |
| 138 | + | NR | NL | IVIG | - | Complete recovery |
| 139 | + | NR | NL | IVIG | - | Complete recovery |
| 140 | - | NR | NL | IVIG | - | Partial recovery |
| 141 | NR | NR | Diffuse enhancement  of the left facial nerve. | IVIG | - | Complete recovery |
| 142 | - | NR | NR | IVIG | - | Complete recovery |
| 143 | + | NR | NL | IVIG | - | Complete recovery |
| 144 | + | - | NL | IVIG | + | Complete recovery |
| 145 | + | NR | + | IVIG  PLEX | - | Partial recovery |
| 146 | + | NR | mild thickening and enhancement of the anterior and posterior spinal nerve roots of the cauda equina | Conservative | - | Complete recovery |
| 147 | + | - | prominent enhancement in the nerve roots of the cauda equina | IVIG | - | Complete recovery |
| 148 | + | - | NR | IVIG | + | Complete recovery |
| 149 | + | - | NL | IVIG | - | Complete recovery |
| 150 | + | NR | NL | IVIG | + | Poor recovery |
| 151 | NR | NR | NL | IVIG  Corticosteroids | - | Partial recovery |
| 152 | + | - | NL | PLEX | + | Partial recovery |
| 153 | + | NR | NL | IVIG | - | Partial recovery |
| 154 | + | NR | NR | IVIG | - | Complete recovery |
| 155 | + | NR | NL | IVIG | - | Complete recovery |
| 156 | NR | NR | NR | IVIG | + | Died |
| 157 | NR | NR | NR | IVIG | - | Complete recovery |
| 158 | NR | Anti GQ1b + | NL | IVIG | - | Complete recovery |
| 159 | + | - | NR | IVIG  Corticosteroids | - | Partial recovery |
| 160 | + | - | NR | IVIG | - | Complete recovery |
| 161 | + | NR | NL | IVIG | - | Partial recovery |
| 162 | + | NR | NR | IVIG  Corticosteroids | - | Complete recovery |
| 163 | NR | NR | NL | IVIG  PLEX | - | Partial recovery |
| 164 | + | Anti GM3 + | NR | IVIG | - | Partial recovery |
| 165 | + | - | NL | IVIG | - | Complete recovery |
| 166 | NR | NR | NR | IVIG | - | Partial recovery |
| 167 | NR | NR | NL | IVIG | - | Partial recovery |
| 168 | NR | NR | NL | IVIG | - | Partial recovery |
| 169 | - | NR | NL | IVIG | - | Partial recovery |
| 170 | - | NR | Spine: mild  enhancement of the ventral roots in the cauda equina | IVIG  PLEX | + | Partial recovery |
| 171 | + | ­- | NR | IVIG | - | Partial recovery |
| **IVIG:** Intravenous Immunoglobulin, **MRI:** Magnetic resonance imaging, **NR:** Not reported, **NL:** Normal, **PLEX:** Plasma exchange, **UK:** United Kingdom, **USA:** United States of America | | | | | | |

| **Supplementary Table 4:** Parsonage turner studies and patients’ characteristics by case. | | | | | | | | | |
| --- | --- | --- | --- | --- | --- | --- | --- | --- | --- |
| **#** | **Author** | **Year** | **Country** | **Sex** | **Age** | **Vaccine type** | **Vaccine dose** | **Time to onset (days)** | **Occurring side** |
| 1 | Vitturi et al. | 2021 | Italy | M | 51 | AstraZeneca | 1^st^ dose | 30 | NR |
| 2 | Shields et al. | 2022 | USA | F | 36 | Pfizer | 1^st^ dose | 7 | Injection side |
| 3 |  |  |  | M | 74 | Pfizer | 2^nd^ dose | 14 | Injection side |
| 4 |  |  |  | M | 50 | Spikevax | 2^nd^ dose | 5 | Injection side |
| 5 |  |  |  | M | 53 | Pfizer | 1^st^ dose | 14 | Injection side |
| 6 |  |  |  | F | 84 | Pfizer | 2^nd^ dose | 56 | Contralateral |
| 7 |  |  |  | F | 46 | Spikevax | 2^nd^ dose | 6 | Injection side |
| 8 | Sharma et al. | 2022 | India | M | 30 | AstraZeneca | 2^nd^ dose | 7 | Injection side |
| 9 | Queler et al. | 2022 | USA | M | 49 | Pfizer | 1^st^ dose | 1 | Bilateral |
| 10 |  |  |  | M | 44 | Spikevax | 2^nd^ dose | 18 | Injection side |
| 11 | Oncel et al. | 2022 | Turkey | M | 56 | Pfizer | 2^nd^ dose | 1 | Injection side |
| 12 | Min et al. | 2022 | South Korea | M | 31 | J&J | 1^st^ dose | 6 | Injection side |
| 13 |  |  |  | M | 37 | J&J | 1^st^ dose | 14 | Injection side |
| 14 |  |  |  | M | 71 | AstraZeneca | 1^st^ dose | 16 | Injection side |
| 15 |  |  |  | M | 63 | AstraZeneca | 1^st^ dose | 14 | Injection side |
| 16 |  |  |  | F | 65 | AstraZeneca | 1^st^ dose | 5 | Injection side |
| 17 |  |  |  | M | 61 | AstraZeneca | 2^nd^ dose | 2 | Injection side |
| 18 |  |  |  | F | 31 | Pfizer | 1^st^ dose | 2 | Injection side |
| 19 |  |  |  | F | 50 | Pfizer | 1^st^ dose | 4 | Contralateral |
| 20 |  |  |  | M | 58 | Pfizer | 1^st^ dose | 5 | Injection side |
| 21 |  |  |  | F | 23 | Pfizer | 1^st^ dose | 10 | Injection side |
| 22 |  |  |  | F | 81 | Pfizer | 1^st^ dose | 15 | Bilateral |
| 23 |  |  |  | M | 39 | Spikevax | 1^st^ dose | 7 | Injection side |
| 24 | Mejri et al. | 2022 | Tunis | M | 50 | Pfizer | 2^nd^ dose | 15 | Injection side |
| 25 | Mahajan et al. | 2021 | USA | M | 50 | Pfizer | 1^st^ dose | 7 | Injection side |
| 26 | Lakkireddy et al. | 2022 | India | M | 21 | AstraZeneca | 1^st^ dose | 7 | Contralateral |
| 27 | Fukahori et al. | 2022 | Japan | F | 14 | Pfizer | 2^nd^ dose | 30 | Injection side |
| 28 | Amjad et al. | 2022 | USA | M | 78 | Pfizer | 2^nd^ dose | 21 | Bilateral |
| 29 | Bernheimer et al. | 2022 | USA | F | 42 | Spikevax | 2^nd^ dose | 21 | Injection side |
| 30 | Civardi et al. | 2022 | Italy | F | 50 | Pfizer | 1^st^ dose | 10 | Injection side |
| 31 | Diaz-Segarra et al. | 2022 | USA | F | 35 | Pfizer | 1^st^ dose | 9 | Contralateral |
| 32 | Crespo et al. | 2021 | Spain | M | 38 | AstraZeneca | 1^st^ dose | 4 | NR |
| 33 | Koh et al. | 2021 | Singapore | M | 50 | Pfizer | 1^st^ dose | 25 | Injection side |
| 34 |  |  | Singapore | M | 44 | Pfizer | 2^nd^ dose | 4 | Contralateral |
| 35 |  |  | Singapore | M | 58 | Spikevax | 2^nd^ dose | 17 | Injection side |
| 36 | Coffman et al. | 2021 | USA | F | 66 | Pfizer | 2^nd^ dose | 14 | Injection side |
| 37 | Flikkema et al. | 2021 | USA | M | 43 | Pfizer | 1^st^ dose | 5 | Injection side |
| 38 | Kim et al. | 2021 | South Korea | F | 45 | AstraZeneca | 1^st^ dose | 2 | Injection side |
| 39 | Chua et al. | 2022 | USA | M | 64 | Spikevax | 2^nd^ dose | 14 | Injection side |
| 40 | James et al. | 2022 | India | F | 70 | AstraZeneca | 1^st^ dose | 2 | Injection side |
| **F:** Female, **J&J:** Johnson and Johnson, **M:** Male, **NR:** Not Reported, | | | | | | | | | |

| **Supplementary Table 5:** Parsonage turner patients’ findings, treatments, and outcomes by case. | | | | | |
| --- | --- | --- | --- | --- | --- |
| # | **Presenting symptoms** | **EMG/NCV** | **Brain MRI** | **Treatments** | **Outcome** |
| 1 | Hypoesthesia and muscle weakness of the upper limb | + | NR | NSAID  Pregabalin | Partial recovery after 5 months |
| 2 | Right sided neck and shoulder pain  Right arm weakness | + | NL | Corticosteroid  Gabapentin | Complete recovery after 3 months |
| 3 | Left forearm pain  Left thumb flexion weakness | + | NL | Physical therapy | Partial recovery after 18 weeks |
| 4 | Right sided neck and shoulder pain  Right arm weakness | + | NL | Corticosteroid  Physical therapy | Complete recovery after 4 months |
| 5 | Left shoulder pain radiating to fingers  Ulnar paresthesia  Hand weakness | + | NL | Corticosteroid  Gabapentin | Partial recovery after 14 weeks |
| 6 | Left forearm pain  Left hand weakness | + | NL | No treatment | Partial recovery after 3 months |
| 7 | Difficulty moving left  shoulder and fingers  Left arm pain | + | NL | Corticosteroid | Significant improvement after corticosteroid injection |
| 8 | Left upper limb weakness | + | NL | NR | NR |
| 9 | Left forearm pain | NL | NL | Corticosteroid | Partial recovery after 2 months |
| 10 | Left deltoid pain  Reduced left shoulder range of motion beyond 20° of abduction | + | NL | Gabapentin | Partial recovery after 5 weeks |
| 11 | Left shoulder pain and muscle weakness | + | NL | NSAIDs | Complete recovery after 3 months |
| 12 | NR | NR | + | No treatment | Complete recovery in one week |
| 13 | NR | NR | NL | Corticosteroid  Gabapentin | Near-complete recovery in 10 weeks |
| 14 | NR | NR | + | Corticosteroid  Gabapentin | Poor recovery in 15 weeks |
| 15 | NR | NR | + | No treatment | Poor recovery in 4 weeks |
| 16 | NR | NR | + | No treatment | Complete recovery within 2 months |
| 17 | NR | NR | + | Corticosteroid | Partial recovery in 5 months |
| 18 | NR | NR | + | No treatment | Complete recovery in 3 weeks |
| 19 | NR | NR | NL | NSAIDs  Fentanyl patch Corticosteroid | Good response to corticosteroid |
| 20 | NR | NR | NR | Corticosteroid  Pregabalin | Poor recovery in 8 weeks |
| 21 | NR | NR | NR | Corticosteroid  Gabapentin | Partial recovery in 6 weeks |
| 22 | NR | NR | NL | pregabalin Nortriptyline  NSAIDs | Poor recovery in 6 months |
| 23 | NR | NR | + | Corticosteroid  Gabapentin | Poor recovery in 8 weeks |
| 24 | Right sided neck and shoulder pain  Right arm weakness | + | NL | Corticosteroid | Partial recovery after 4 weeks |
| 25 | Weakness of left-hand grip and wrist extension | + | NL | Corticosteroid | Complete recovery after 5 weeks |
| 26 | Right shoulder pain and weakness | + | NL | Corticosteroid  Pregabalin | Complete recovery after 10 weeks |
| 27 | Acute sharp pain in the left posterior region of the neck | NL | + | IVIG | Partial recovery 4 days after IVIG |
| 28 | Bilateral hand weakness | + | NL | Corticosteroid | Partial recovery at discharge |
| 29 | Severe left shoulder pain and paresthesia radiating to the left upper extremity | + | NL | Corticosteroid  Gabapentin | Complete recovery after 2 months |
| 30 | Severe left arm pain, specifically on the shoulder and elbow. | + | NL | Corticosteroid  pregabalin | NR |
| 31 | Left arm weakness, numbness, and paresthesia | NL | NR | Corticosteroid | Complete recovery after 6 weeks |
| 32 | Left shoulder pain, radiating to the scapular region and arm | NR | NL | Corticosteroid | Partial recovery after 2 weeks |
| 33 | Right arm weakness, numbness, and paresthesia | NL | + | Corticosteroid | Complete recovery after 7 weeks |
| 34 | Right sided neck and shoulder pain  Right arm, forearm, and weakness  Right forearm and hand paresthesia | + | NL | No treatment | Complete recovery after 2 months |
| 35 | Left shoulder and arm pain  Left hand paresthesia and weakness | + | NR | Corticosteroid | Complete recovery after 5 weeks |
| 36 | Persistent right shoulder dysfunction | + | NL | Physical therapy | Partial recovery after 3 months |
| 37 | Right shoulder pain  Right upper extremity weakness | NR | NL | Corticosteroid | NR |
| 38 | Acute left leg paralysis  and paresthesia | + | NL | Corticosteroid | Poor recovery after 1 month |
| 39 | Intense, unremitting left shoulder pain and muscle tenderness | + | + | Corticosteroid | Partial recovery after 1 month |
| 40 | Right upper arm, shoulder, and neck pain | + | + | NR | Poor recovery after 1 month |
| **IVIG:** Intravenous Immunoglobulin, **NL:** Normal, **NR:** Not Reported, **PT:** Physical Therapy | | | | | |

| **Supplementary table 6.** Facial nerve palsy patients’ findings, treatments, and clinical outcomes by case. | | | | | | | | | | | | |
| --- | --- | --- | --- | --- | --- | --- | --- | --- | --- | --- | --- | --- |
| **#** | **Author** | **Country** | **Year** | **Sex** | **Age** | **Vaccine type** | **Vaccine dose** | **Time to onset (days)** | **Prior history of FNP** | **Presenting symptoms** | **Treatment** | **Outcome** |
| 1 | Repajic et al. | USA | 2021 | F | 57 | Pfizer | 2^nd^ dose | 3 | 3 times | Bilateral FW | Corticosteroid  Viral agents | Improved after 2 weeks |
| 2 | Poudel et al. | USA | 2022 | F | 17 | Spikevax | 1^st^ dose | 1 | - | Left-sided FW | Corticosteroid | Improved after 2 weeks |
| 3 | Colella et al. | Italy | 2021 | M | 37 | Pfizer | 1^st^ dose | 3 | - | Left-sided FW | Corticosteroid | Improved after 2 weeks |
| 4 | Pothiawala et al. | Singapore | 2021 | M | 46 | Spikevax | 2^nd^ dose | 10 | - | Right-sided FW | Corticosteroid Acyclovir | NR |
| 5 | Iftikhar et al. | Qatar | 2021 | M | 36 | Spikevax | 2^nd^ dose | 1 | - | Left sided FW | Corticosteroid | Improved after 2 weeks |
| 6 | Zhang et al. | USA | 2022 | F | 38 | Pfizer | 1^st^ dose | 6 | 10 years earlier | Left-sided FW | Corticosteroid | Partially improved after 7 days |
| 7 | Mirmosayyeb et al. | Iran | 2022 | F | 27 | Sputnik V | 1^st^ dose | 3 | - | Left-sided FW | Corticosteroid Valacyclovir | Improved after 10 days |
| 8 |  |  | 2022 | M | 58 | Sputnik V | 1^st^ dose | 6 | - | Left-sided FW | Corticosteroid  Valacyclovir | Improved after 1 week |
| 9 | Nishizawa et al. | Japan | 2021 | F | 62 | J&J | 1^st^ dose | 18 | - | Right-sided FW | NR | NR |
| 10 | Cellina et al. | Italy | 2022 | F | 35 | Spikevax | 1^st^ dose | 1 | - | Left-sided FW | Corticosteroid | Improved after 19 days |
| 11 | Martin-villares et al. | Spain | 2022 | F | 34 | Spikevax | 1^st^ dose | 2 | 10 years earlier | Right-sided FW | Corticosteroid | Improved between 10 - 22 days |
| 12 | Obermann et al. | Germany | 2021 | F | 21 | Pfizer | 1^st^ dose | 2 | - | Right-sided FW | Corticosteroid | Lower facial muscles improved after 3 days |
| 13 | Yu et al. | China | 2021 | F | 36 | Sinovac | 1^st^ dose | 2 | 19 years earlier | Right sided FW | Corticosteroid  Fluorometholone drop | Improved after 2 weeks |
| 14 | Burrows et al. | UK | 2021 | M | 61 | Pfizer | 1^st^ dose | 2 | - | Bilateral FW | Corticosteroid | Improved after 2 weeks |
| 15 | Mussatto et al. | USA | 2022 | M | 60 | Pfizer | 1^st^ dose | 2 | - | Left-sided FW | Corticosteroid  Valacyclovir | Improved after 90 days |
| 16 | Ish et al. | India | 2021 | M | 50 | Covaxin | 2^nd^ dose | 3 | - | Right-sided FW | Corticosteroid  Topical antibiotic | Improved after 10 days |
| 17 | Salbas et al. | Turkey | 2021 | F | 33 | Pfizer | 1^st^ dose | 4 | - | Left-sided FW | Corticosteroid | Improved after 4 weeks |
| 18 |  |  |  | M | 49 | Pfizer | 1^st^ dose | 10 | - | Right-sided FW | Corticosteroid | Improved after 3 weeks |
| 19 | Galimi et al. | Italy | 2021 | F | 54 | Pfizer | 1^st^ dose | 1 | - | Left sided FW | Corticosteroid  Famciclovir | Improved after 4 weeks |
| **F:** Female, **FNP:** Facial Nerve Palsy, **FW:** Facial weakness, **J&J:** Johnson and Johnson, **M:** Male, **UK:** United Kingdom, **USA:** United States of America | | | | | | | | | | | | |

| **Supplementary table 7.**  Small fiber neuropathy and tolosa-hunt patients’ findings, treatments, and clinical outcomes by case. | | | | | | | | | | | | |  |
| --- | --- | --- | --- | --- | --- | --- | --- | --- | --- | --- | --- | --- | --- |
| **#** | **author** | **Country** | **Year** | **Sex** | **Age** | **Vaccine type** | **Time to onset (day)** | **Vaccine dose** | **Presenting symptoms** | | **Skin punch biopsy** | **Treatment** | **Outcome** |
| ***Small fiber neuropathy patients*** | | | | | | | | | | | | | |
| 1 | Finsterer et al. | Austria | 2022 | F | 40 | Pfizer | 10 | 2^nd^ dose | Muscle weakness  Gait disturbance | | Reduced IENFD | Diltiazem  Loratadine  Steroids  IVIG | IVIG had beneficial effects |
| 2 |  |  |  | F | 52 | Spikevax | 17 | 2^nd^ dose | Dysautonomia  (Balance problems, dysphagia) | | NR | No treatment | Symptoms resolved spontaneously |
| 3 |  |  |  | F | 32 | Pfizer | 1 | 2^nd^ dose | Paresthesia  Muscle weakness | | Reduced IENFD | Symptom treatment | Resolved |
| 4 | Waheed et al. | USA | 2021 | F | 57 | Pfizer | 7 | 2^nd^ dose | Burning  Dysesthesias in the feet | | Reduced IENFD above the lateral malleolus | Gabapentin | Improved completely in 2 weeks |
| 5 | Khokhar et al. | USA | 2022 | F | 64 | Spikevax | 21 | Booster | Paroxysmal tingling affecting mainly the feet, Left > Right | | Length-dependent neuropathy affecting unmyelinated sensory fibers of the thigh | NR | NR |
| ***Tolosa hunt patients*** | | | | | | | | **Presenting symptoms** | | **Imaging** | | **Treatment** | **Outcome** |
| 1 | Chauang et al. | USA | 2021 | M | 45 | Spikevax | 14 | Sever left sided headache, periorbital pain, and ptosis  Binocular diplopia  Decreased visual acuity | | *Brain CT:* concerning for cavernous sinus thrombosis versus dural-based mass.  *Brain MRI:* bilateral perineural enhancement surrounding the optic nerve sheaths (L>R) | | Steroid | Symptoms improved after 2 months |
| **F:** Female, **ICH:** Intracranial Hemorrhage, **IENFD:** Intraepidermal Nerve Fiber Density, **J&J:** Johnson and Johnson, **M:** Male, **NR:** Not reported **R:** Not reported | | | | | | | | | | | | | |

| **Supplementary Table 8:** Myasthenia gravis studies and patients’ characteristics by case. | | | | | | | | | |
| --- | --- | --- | --- | --- | --- | --- | --- | --- | --- |
| **#** | **Author** | **Country** | **Year** | **Sex** | **Age** | **Vaccine type** | **Vaccine dose** | **Time to onset (days)** | **MG type** |
| 1 | Abicic et al. | Croatia | 2022 | M | 65 | Pfizer | Booster | 21 | Oculobulbar |
| 2 | Chavez et al. | USA | 2021 | M | 82 | Pfizer | 2^nd^ dose | 2 | Generalized |
| 3 | Fanella et al. | Italy | 2022 | M | 90 | Pfizer | 2^nd^ dose | 10 | Oculobulbar |
| 4 |  |  |  | M | 80 | AstraZeneca | 2^nd^ dose | 6 | Oculobulbar |
| 5 |  |  |  | M | 55 | Spikevax | 1st dose^a^ | 3 | Generalized |
| 6 | Hoshina et al. | Japan | 2022 | M | 30 | Spikevax | 1^st^ dose | 2 | Oculobulbar |
| 7 | Kang et al. | S. Korea | 2022 | M | 35 | AstraZeneca | 1^st^ dose | 7 | Oculobulbar |
| 8 | Lee et al. | S. Korea | 2021 | F | 33 | Pfizer | 2^nd^ dose | 1 | Generalized |
| 9 | Slavin et al. | USA | 2022 | M | 60 | Spikevax | Booster | 6 | Generalized |
| 10 | Ramdas et al. | UK | 2022 | F | 13 | Pfizer | 1^st^ dose | 14 | Generalized |
| 11 |  |  |  | M | 59 | AstraZeneca | 1^st^ dose | 2 | Generalized |
| 12 |  |  |  | M | 63 | Pfizer | Booster | 3 | Oculobulbar |
| 13 |  |  |  | M | 73 | Pfizer | Booster | 12 | Generalized |
| 14 |  |  |  | M | 50 | Pfizer | 1^st^ dose | 7 | Oculobulbar |
| 15 |  |  |  | F | 83 | Pfizer | 1^st^ dose | 6 | Generalized |
| 16 |  |  |  | M | 77 | AstraZeneca | 1^st^ dose | 3 | Generalized |
| 17 | Maher et al. | Australia | 2022 | M | 52 | AstraZeneca | 1^st^ dose | 1 | Oculobulbar |
| 18 | Galassi et al. | Italy | 2022 | M | 73 | AstraZeneca | 1^st^ dose | 8 | Oculobulbar |
| 19 | Watad et al. | Israel | 2021 | M | 72 | Pfizer | 2^nd^ dose | 1 | Generalized |
| 20 |  |  |  | M | 73 | Pfizer | 2^nd^ dose | 7 | Generalized |
| 21 | Virgilio et al. | Italy | 2022 | M | 73 | AstraZeneca | 1^st^ dose | 28 | Generalized |
| 22 | Devaraj et al. | India | 2022 | F | 50 | AstraZeneca | 1^st^ dose | 7 | Oculobulbar |
| **F:** Female, **M:** Male, **MG:** Myasthenia Gravis, **UK:** United Kingdom, **USA:** United States of America.  **a:** Symptoms worsened after the second dose | | | | | | | | | |

| **Supplementary Table 9:** Myasthenia gravis patients’ findings, treatments, and clinical outcomes by case. | | | | | | | |
| --- | --- | --- | --- | --- | --- | --- | --- |
| **#** | **Presenting symptoms** | **Imaging** | **EMG** | **Seropositivity** | **Other findings** | **Treatment** | **Outcome** |
| 1 | Binocular diplopia | Brain CT: NL  Brain MRI: Non-specific | NR | AChR +  MuSK - | IM neostigmine test - | Pyridostigmine  Prednisolone | Improved after one month |
| 2 | Intermittent dysarthria | Brain CT: NL  Brain MRI: NL  Thymus hyperplasia: - | RNS + | AChR + | NR | Pyridostigmine  Speech therapy | Initial improvement after 2 weeks  Myasthenic crisis after two months  Recovered and is rehabilitating |
| 3 | Asthenia, Bilateral ptosis | Thymus hyperplasia: - | RNS + | AChR + | NR | Pyridostigmine | Symptoms unchanged after one month |
| 4 | Binocular diplopia, Bilateral ptosis, dysphagia | Thymus hyperplasia: - | RNS + | AChR + | NR | Pyridostigmine  Prednisolone  Azathioprine  Plasma exchange | Improved after 2^nd^ plasma exchange  Mild ptosis at 3 months f/u |
| 5 | Upper limbs fatigability Binocular diplopia | NR | RNS + | AChR + | NR | Pyridostigmine  IVIG | At discharge: ptosis after 50s of upward gaze  At 3-month f/u: upper limb fatigability after 90s of abduction |
| 6 | Binocular diplopia | Brain CT: NL  Brain MRI: NL | NR | AChR +  MuSK - | Iced-pack test +  IM neostigmine test + | Pyridostigmine  Prednisolone | Symptoms improved but continue to fluctuate |
| 7 | Binocular diplopia | Brain MRI: NL  Thymus hyperplasia: - | NR | AChR + | NR | NR | NR |
| 8 | Bilateral ptosis, Binocular diplopia | Thymus hyperplasia: Mild | RNS + | AChR -  MuSK - | IM neostigmine test + | Pyridostigmine | Partially improved after 4 days |
| 9 | Dysarthria, Binocular diplopia dysphagia | Brain CT: NL  Thymus hyperplasia: - | RNS + | AChR - | NR | Pyridostigmine | Symptoms improved |
| 10 | NR | NR | RNS + | AChR - | NR | Pyridostigmine  Prednisolone | NR |
| 11 | NR | NR | NR | AChR + | NR | Pyridostigmine  Prednisolone | NR |
| 12 | NR | NR | NR | AChR + | NR | Pyridostigmine | NR |
| 13 | NR | NR | SFEMG + | AChR + | NR | Pyridostigmine  Prednisolone  IVIG | NR |
| 14 | NR | NR | RNS - | AChR + | NR | Pyridostigmine | NR |
| 15 | NR | NR | RNS - | AChR + | NR | Pyridostigmine  Prednisolone  IVIG | NR |
| 16 | NR | NR | RNS +  SFEMG + | AChR + | NR | Pyridostigmine  Prednisolone  Plasma exchange | NR |
| 17 | Binocular diplopia | Brain MRI: NL | SFEMG + | AChR -  MuSK - | Iced-pack test + | Pyridostigmine  Prednisolone | Partially improved |
| 18 | Left-sided ptosis | Brain CT: NL | RNS + | AChR + | NR | Pyridostigmine | Symptoms improved |
| 19 | NR | NR | RNS + | NR | NR | Prednisolone  Plasma exchange | Rapid response to treatment with quick symptom improvement |
| 20 | NR | NR | RNS +  SFEMG + | NR | NR | Pyridostigmine  Plasma exchange | Intubated due to respiratory failure |
| 21 | Left-sided ptosis  Binocular diplopia | Brain MRI: NL  Thymoma: + | RNS + | AChR + | NR | Pyridostigmine  Prednisolone  Azathioprine | Partially improved during 16 days of hospitalization  Complete improvement at follow-up |
| 22 | Bilateral ptosis  Binocular diplopia  Dysarthria Dysphagia | Thymus hyperplasia: - | RNS + | AChR + | IM neostigmine test + | Pyridostigmine  Prednisolone  Mycophenolate mofetil | Partially improved during 7 days of hospitalization  Complete improvement at follow-up |
| **AChR:** Acetylcholine Receptor antibody, **CT:** Computed Tomography, **EMG:** Electromyography, **IM:** Intramuscular, **IVIG:** Intravenous Immunoglobulin, **MRI:** Magnetic Resonance Imaging, **MuSK:** Muscle Specific Kinase antibody, **NL:** Normal, **NR:** Not Reported, **RNS:** Repetitive Nerve Stimulation, **SFEMG:** Single Fiber Electromyography | | | | | | | |
